# Polymorphisms within Novel Risk Loci for Type 2 Diabetes Determine β-Cell Function

**DOI:** 10.1371/journal.pone.0000832

**Published:** 2007-09-05

**Authors:** Harald Staiger, Fausto Machicao, Norbert Stefan, Otto Tschritter, Claus Thamer, Konstantinos Kantartzis, Silke A. Schäfer, Kerstin Kirchhoff, Andreas Fritsche, Hans-Ulrich Häring

**Affiliations:** Department of Internal Medicine, Division of Endocrinology, Diabetology, Angiology, Nephrology, and Clinical Chemistry, Eberhard-Karls-University Tübingen, Tübingen, Germany; Mayo Clinic College of Medicine, United States of America

## Abstract

**Background:**

Type 2 diabetes arises when insulin resistance-induced compensatory insulin secretion exhausts. Insulin resistance and/or β-cell dysfunction result from the interaction of environmental factors (high-caloric diet and reduced physical activity) with a predisposing polygenic background. Very recently, genetic variations within four novel genetic loci (*SLC30A8*, *HHEX*, *EXT2*, and *LOC387761*) were reported to be more frequent in subjects with type 2 diabetes than in healthy controls. However, associations of these variations with insulin resistance and/or β-cell dysfunction were not assessed.

**Methodology/Principal Findings:**

By genotyping of 921 metabolically characterized German subjects for the reported candidate single nucleotide polymorphisms (SNPs), we show that the major alleles of the *SLC30A8* SNP rs13266634 and the *HHEX* SNP rs7923837 associate with reduced insulin secretion stimulated by orally or intravenously administered glucose, but not with insulin resistance. In contrast, the other reported type 2 diabetes candidate SNPs within the *EXT2* and *LOC387761* loci did not associate with insulin resistance or β-cell dysfunction, respectively.

**Conclusions/Significance:**

The *HHEX* and *SLC30A8* genes encode for proteins that were shown to be required for organogenesis of the ventral pancreas and for insulin maturation/storage, respectively. Therefore, the major alleles of type 2 diabetes candidate SNPs within these genetic loci represent crucial alleles for β-cell dysfunction and, thus, might confer increased susceptibility of β-cells towards adverse environmental factors.

## Introduction

Type 2 diabetes mellitus (T2DM) reaches epidemic dimensions in western industrialized nations and is caused by environmental factors, such as high-caloric fat- and carbohydrate-enriched diets and a sedentary lifestyle with markedly reduced physical activity. Moreover, as one of the most recognized polygenic diseases, T2DM is due to variations within several genetic loci that confer increased susceptibility towards the above mentioned environmental challenges [1]. Linkage studies, candidate-gene approaches, and genome-wide association studies identified single nucleotide polymorphisms (SNPs) within currently up to ten genes which associate with an increased T2DM risk. During the pathogenesis of T2DM, insulin resistance of peripheral tissues (liver, skeletal muscle, and adipose tissue) provokes compensatory increments in insulin secretion by pancreatic β-cells. When insulin resistance is no longer compensated and β-cells exhaust, hyperglycemia arises [2]. Thus, most T2DM risk loci are supposed to contribute to β-cell dysfunction. In fact, among the most prominent T2DM risk loci up to now, only SNPs within *PPARG* [3]–[5] contribute to altered insulin sensitivity, whereas SNPs within *KCNJ11* [6]–[8], *CDKAL1* [9], and *TCF7L2* [10]–[13] impair β-cell function.

In the first very recently reported genome-wide association study for T2DM [14], four novel T2DM risk loci were identified. The role of the corresponding genes, i.e. *SLC30A8*, *HHEX*, *EXT2*, and *LOC387761*, in the development of prediabetes phenotypes was not assessed and is not established in the literature. Therefore, it was the aim of the present study to test the association of the recently identified candidate SNPs within or near the genes *SLC30A8*, *HHEX*, *EXT2*, and *LOC387761* with insulin resistance and β-cell dysfunction in a thoroughly metabolically characterized German population at an increased risk for T2DM.

## Methods

### Subjects

One thousand non-diabetic subjects were recruited from the southern part of Germany and participated in the ongoing Tübingen Family Study for T2DM (TÜF) which currently includes ∼2000 individuals. Recruitment of the subjects was based on (i) exclusion of subjects with anti-glutamic acid decarboxylase antibodies, impaired glucose tolerance, and T2DM as well as (ii) inclusion of subjects of whom DNA samples and C-peptide measurements were available. From the 1000 subjects selected in this way, 79 subjects were excluded due to incomplete data sets. 71 % of the subjects had a recorded family history of T2DM, i.e. at least one 2^nd^-degree relative with T2DM. All participants underwent the standard procedures of the protocol including medical history and physical examination, assessment of smoking status, alcohol consumption habits and activity, routine blood tests, and oral glucose tolerance test (OGTT). A subgroup of 491 subjects voluntarily agreed to undergo a hyperinsulinemic-euglycemic clamp. Another subgroup of the clamped subjects (N = 150) additionally agreed to undergo an intravenous glucose tolerance test (IVGTT). The participants were not taking any medication known to affect glucose tolerance or insulin secretion. The participants gave informed written consent to the study, and the protocol was approved by the local ethical committee (Ethik-Kommission der Medizinischen Fakultät der Universität Tübingen).

### Genotyping of the study population

For genotyping, DNA was isolated from whole blood using a commercial DNA isolation kit (NucleoSpin, Macherey & Nagel, Düren, Germany). SNPs were genotyped using the TaqMan assay (Applied Biosystems, Foster City, CA, USA). The TaqMan genotyping reaction was amplified on a GeneAmp PCR system 7000 (50°C for 2 min, 95°C for 10 min, followed by 40 cycles of 95°C for 15 s and 60°C for 1 min), and fluorescence was detected on an ABI Prism sequence detector (Applied Biosystems, Foster City, CA, USA).

### Body composition and body fat distribution

Body composition was measured by bioelectrical impedance as the percentage of body fat. Body mass index (BMI) was calculated as weight divided by the square of height (kg/m^2^). Waist circumference was measured in the upright position at the midpoint between the lateral iliac crest and the lowest rib.

### OGTT

After a 10-h overnight fast, all subjects underwent a 75-g OGTT and venous blood samples were obtained at 0, 30, 60, 90, and 120 min for determination of plasma glucose, insulin, and C-peptide.

### Hyperinsulinemic-euglycemic clamp and IVGTT

After an overnight fast and a 60-min. baseline period, 491 subjects received a priming dose of insulin followed by an infusion (40 mU/m^2^) of short-acting human insulin for 120 min. A variable infusion of 20 % (w/v) glucose was started to maintain the plasma glucose concentration at 5.5 mM. Blood samples for the measurement of plasma glucose were obtained at 5-min. intervals throughout the clamp. Plasma insulin levels were measured at baseline and in the steady state of the clamp. In a subgroup of the clamped subjects (N = 150), an IVGTT was performed prior to the clamp, as described by the Botnia protocol [15]. After baseline samples had been collected, a 0.3 g/kg body weight glucose dose of a 20 % (w/v) glucose solution was given at time 0. Blood samples for the measurement of plasma glucose, insulin, and C-peptide were obtained at 2, 4, 6, 8, 10, 20, 30, 40, 50, and 60 min.

### Determination of blood parameters

Plasma glucose was determined using a bedside glucose analyzer (glucose oxidase method, Yellow Springs Instruments, Yellow Springs, CO, USA). Plasma insulin levels were determined by microparticle enzyme immunoassay (Abbott Laboratories, Tokyo, Japan), and plasma C-peptide by radioimmunoassay (Byk-Sangtec, Dietzenbach, Germany).

### Calculations

The area under the curve (AUC) of plasma glucose levels during OGTT was calculated as 0.5·(0.5·Glc_0_+Glc_30_+Glc_60_+Glc_90_+0.5·Glc_120_). The AUC of plasma C-peptide levels during OGTT was calculated analogously. Insulin secretion in the OGTT was assessed by calculating the AUC of C-peptide divided through the AUC of glucose (AUC C-pep/AUC glc). First-phase insulin secretion (in nM) was estimated from plasma insulin and glucose concentrations during OGTT using validated equations as described formerly [16]. Homeostasis model assessment of insulin resistance (HOMA-IR, in arbitrary units, U) was calculated as (2·Glc_0_·Ins_0_)/45. Insulin sensitivity from OGTT (in arbitrary units, U) was estimated as proposed by Matsuda and DeFronzo [17]: 10,000/(Glc_0_·Ins_0_·Glc_mean_·Ins_mean_)^½^. Clamp-derived insulin sensitivity (in arbitrary units, U) was calculated as glucose infusion rate necessary to maintain euglycemia during the last 40 min. (steady state) of the clamp (in µmol·kg^−1^·min^−1^) divided by the steady-state insulin concentration.

### Statistical analyses

Unless otherwise stated, the data are given as means±SE. Hardy-Weinberg equilibrium was tested using χ^2^ test. Simple and multivariate linear regression analyses were carried out after log-transformation of data followed by ANOVA. In multivariate linear regression models, the trait (index of insulin sensitivity/secretion) was chosen as dependent variable. Two-group comparisons were performed using Student's t-test. Differences between time courses were tested by MANOVA for repeated measures. A p-value<0.05 was considered statistically significant. In our cohort of 921 subjects, we were able to detect an effect size (δ) of 1/5 standard deviation (σ) of a quantitative trait with a power of 100 % in the additive as well as the dominant model. Effect sizes of 1/10 σ were still detected with 78 % power in the additive model and with 86 % power in the dominant model. The statistical software package JMP 4.0 (SAS Institue, Cary, NC, USA) was used.

## Results

We genotyped 921 non-diabetic subjects (clinical characteristics given in Table 1) for the three intronic SNPs rs3740878, rs11037909, and rs1113132 of the *EXT2* gene (chr. 11), for the two SNPs rs1111875 and rs7923837 in the 3′-flanking region of the *HHEX* gene (chr. 10), for the non-synonymous SNP rs13266634 (R325W) in the final exon of the *SLC30A8* gene (chr. 8), and for SNP rs7480010 in the 5′-flanking region of the hypothetical gene *LOC387761* (chr. 11). During genotyping of the first 80 subjects, the three *EXT2* SNPs revealed complete genetic linkage consistent with recent phase II data of the International HapMap Project derived from Utah residents with ancestry from northern and western Europe (release #21a January 2007, http://www.hapmap.org/index.html.en, D′ = 1.0, r^2^ = 1.0 for all three SNP pairs). Therefore, among the *EXT2* SNPs, rs11037909 was arbitrarily chosen as representative and further analysed. According to HapMap data, both SNPs located within the *HHEX* locus were not in complete linkage disequilibrium (D′ = 0.959, r^2^ = 0.698) and therefore analysed separately in the total study population. The five remaining completely analysed SNPs were in Hardy-Weinberg equilibrium (p>0.4, all) and displayed minor allele frequencies (MAFs) similar to those recently reported [14] (Tables 2 and 3). The overall genotyping success rate was 99.98 %, and rescreening of 3.16 % of subjects gave 100 % identical results.

**Table 1:** Clinical characteristics of the study population (N = 921).

	women (N = 571)		men (N = 350)	
	mean±SE	range	mean±SE	range
Age (y)	38.6±0.5	15–69	39.0±0.7	18–69
BMI (kg/m^2^)	29.7±0.4	16.3–68.5	28.5±0.4	18.7–67.2
Body fat (%)	35.0±0.4	9.0–63.7	23.1±0.4	6.8–62.0
Waist circumference (cm)	91.6±0.7	56–178	99.2±1.0	52–183
Fasting glucose (mM)	5.07±0.02	3.00–7.17	5.16±0.03	3.50–7.42
Glucose 120 min. OGTT (mM)	6.45±0.07	2.44–11.06	6.09±0.09	2.67–11.06
Fasting insulin (pM)	66.9±2.3	11.0–614.0	59.2±2.7	11.0–362.0
Insulin 120 min. OGTT (pM)	465±17	43–3477	393±24	22–4351

As presented in Tables 2 and 3, none of the SNPs significantly correlated with anthropometric data, such as gender, age, BMI, or body fat content. Only the minor allele of the *SLC30A8* SNP rs13266634 significantly correlated with elevated waist circumference (p = 0.0366) and showed a trend towards a correlation with higher BMI (P = 0.0507) pointing to an association with abdominal adiposity (Table 2). Moreover, the minor allele of the same SNP displayed a significant correlation with lower insulin sensitivity, as measured by the hyperinsulinemic-euglycemic clamp (p = 0.0077) which however disappeared after adjustment for gender, age, and BMI (significant determinants of insulin sensitivity) (Table 2). None of the other SNPs was significantly associated with indices of insulin sensitivity derived from OGTT or hyperinsulinemic-euglycemic clamp (Tables 2 and 3). With different OGTT-derived measures of insulin secretion, no reliable associations of *EXT2* SNP rs11037909, *SLC30A8* SNP rs13266634, and *LOC387761* SNP rs7480010 were detected (Table 2). However, the major alleles of both *HHEX* SNPs were significantly correlated with reduced insulin secretion, as estimated from all three OGTT-derived indices of insulin secretion, even after adjustment for gender, age, BMI, and OGTT-derived insulin sensitivity (significant determinants of insulin secretion) (Table 3). Adjustment for fasting plasma glucose, instead of OGTT-derived insulin sensitivity, resulted in very similar results (data not shown).

**Table 2:** Correlations of *EXT2* SNP rs11037909, *SLC30A8* SNP rs13266634, and *LOC387761* SNP rs7480010 with insulin sensitivity and insulin secretion.

SNP (MAF) Genotype	*EXT2* rs11037909 (0.253)			p_1_	p_2_	p_3_	*SLC30A8* rs13266634 (0.284)			p_1_	p_2_	p_3_	*LOC387761* rs7480010 (0.285)			p_1_	p_2_	p_3_
	TT	TC	CC				CC	CT	TT				AA	AG	GG			
N	506	364	51	-	-	-	480	358	83	-	-	-	463	390	67	-	-	-
Age (y)	38.8±0.6	38.9±0.7	37.6±1.8	0.7	-	-	38.5±0.6	39.3±0.7	38.0±1.4	0.4	-	-	39.0±0.6	38.6±0.6	38.7±1.5	1.0	-	-
BMI (kg/m^2^)	29.3±0.4	29.2±0.4	28.9±1.2	0.9	-	-	28.6±0.4	29.9±0.4	29.9±0.9	0.05	-	-	29.2±0.4	29.0±0.4	31.2±1.0	0.25	-	-
Body fat (%)	30.5±0.5	30.8±0.6	29.0±1.5	0.4	-	-	30.2±0.5	30.8±0.6	30.8±1.2	0.6	-	-	30.6±0.5	30.3±0.6	31.0±1.4	0.7	-	-
Waist circum-ference (cm)	94.6±0.8	94.6±1.0	92.4±2.6	0.6	-	-	93.1±0.8	95.9±1.0	96.7±2.0	0.0366	-	-	94.6±0.8	94.1±0.9	96.8±2.3	0.7	-	-
Fasting glucose (mM)	5.10±0.03	5.11±0.03	5.18±0.08	0.7	0.4	0.5	5.11±0.03	5.11±0.03	5.03±0.06	0.6	0.12	0.06	5.13±0.03	5.07±0.03	5.13±0.07	0.23	0.3	0.12
Glucose 120 min. OGTT (mM)	6.33±0.08	6.26±0.09	6.51±0.24	0.8	0.4	0.9	6.29±0.08	6.38±0.09	6.15±0.19	0.6	0.7	0.7	6.34±0.08	6.22±0.09	6.65±0.21	0.18	0.3	0.7
ISI, OGTT (U)	16.2±0.5	16.5±0.6	15.9±1.5	1.0	1.0	1.0	16.9±0.5	15.7±0.6	15.7±1.2	0.10	0.7	0.5	15.9±0.5	17.1±0.5	14.2±1.3	0.09	0.20	0.17
ISI, clamp (U)*	0.085±0.003	0.088±0.004	0.085±0.011	1.0	0.9	0.6	0.094±0.003	0.078±0.004	0.078±0.008	0.0077	0.5	0.23	0.083±0.004	0.090±0.004	0.084±0.010	0.4	0.6	1.0
HOMA-IR (U)	2.54±0.10	2.46±0.12	2.36±0.32	0.9	1.0	0.8	2.34±0.10	2.72±0.12	2.42±0.25	0.16	0.8	0.7	2.53±0.11	2.41±0.12	2.83±0.28	0.18	0.4	0.21
1^st^-phase insulin secretion (nM)	1.29±0.04	1.33±0.04	1.09±0.12	0.24	0.0287	0.8	1.23±0.04	1.35±0.05	1.42±0.09	0.12	0.4	0.3	1.32±0.04	1.25±0.04	1.35±0.10	0.4	1.0	0.9
C-peptide 30 min. OGTT (nM)	2.08±0.04	2.08±0.05	1.98±0.13	0.9	0.6	0.6	2.00±0.04	2.14±0.05	2.23±0.10	0.05	0.23	0.21	2.13±0.04	2.01±0.05	2.05±0.11	0.07	0.08	0.05
AUC C-pep/AUC glc (pM/mM)	325±5	321±6	302±15	0.3	0.21	0.13	314±5	329±6	334±12	0.20	0.4	0.23	328±5	314±6	325±13	0.07	0.22	0.08

For statistical analysis, data were log-transformed. Plasma glucose levels and indices of insulin sensitivity were adjusted for gender, age, and BMI. Indices of insulin secretion were adjusted for gender, age, BMI, and ISI (OGTT). p_1_–unadjusted; p_2_–adjusted, additive model; p_3_ – adjusted, dominant model. AUC – area under the curve; HOMA-IR – homeostasis model assessment of insulin resistance; ISI – insulin sensitivity index; MAF – minor allele frequency; SNP – single nucleotide polymorphism. *subgroup (N = 491).

**Table 3:** Correlations of *HHEX* SNPs rs1111875 and rs7923837 with insulin sensitivity and insulin secretion.

SNP (MAF) Genotype	*HHEX* rs1111875 (0.401)			p_1_	p_2_	p_3_	*HHEX* rs7923837 (0.367)			p_1_	p_2_	p_3_
	GG	GA	AA				GG	GA	AA			
N	327	449	145	-	-	-	364	438	119	-	-	-
Age (y)	39.2±0.7	38.2±0.6	39.5±1.0	0.5	-	-	39.2±0.7	38.3±0.6	39.0±1.2	0.5	-	-
BMI (kg/m^2^)	29.0±0.5	29.5±0.4	28.9±0.7	0.7	-	-	29.4±0.4	29.1±0.4	29.1±0.8	0.8	-	-
Body fat (%)	30.9±0.6	30.5±0.5	29.7±0.9	0.4	-	-	31.1±0.6	30.0±0.5	30.2±1.0	0.4	-	-
Waist circum-ference (cm)	94.4±1.0	95.4±0.9	92.1±1.5	0.24	-	-	95.1±1.0	94.5±0.9	92.8±1.7	0.5	-	-
Fasting glucose (mM)	5.10±0.03	5.11±0.03	5.08±0.05	0.8	0.7	0.9	5.11±0.03	5.11±0.03	5.08±0.05	0.9	0.7	0.7
Glucose 120 min. OGTT (mM)	6.43±0.09	6.26±0.08	6.22±0.14	0.4	0.3	0.15	6.49±0.09	6.22±0.08	6.11±0.16	0.0494	0.05	0.0321
ISI, OGTT (U)	15.9±0.6	16.5±0.5	16.4±0.9	0.7	0.7	0.4	15.9±0.6	16.7±0.5	16.0±1.0	0.9	0.9	0.9
ISI, clamp (U)*	0.079±0.004	0.091±0.004	0.088±0.006	0.4	0.6	0.4	0.081±0.004	0.090±0.004	0.090±0.007	0.5	0.4	0.6
HOMA-IR (U)	2.42±0.13	2.60±0.11	2.32±0.19	0.6	0.7	0.7	2.47±0.12	2.54±0.11	2.43±0.21	1.0	1.0	0.9
1^st^-phase insulin secretion (nM)	1.22±0.05	1.35±0.04	1.29±0.07	0.07	0.0155	0.0039	1.22±0.04	1.34±0.04	1.35±0.08	0.10	0.0066	0.0023
C-peptide 30 min. OGTT (nM)	1.96±0.05	2.14±0.04	2.10±0.08	0.0423	0.0088	0.0021	1.98±0.05	2.13±0.04	2.14±0.08	0.14	0.0255	0.0075
AUC C-pep/AUC glc (pM/mM)	304±6	331±5	334±9	0.0027	0.0010	0.0002	307±6	329±5	341±10	0.0340	0.0136	0.0122

For statistical analysis, data were log-transformed. Plasma glucose levels and indices of insulin sensitivity were adjusted for gender, age, and BMI. Indices of insulin secretion were adjusted for gender, age, BMI, and ISI (OGTT). p_1_ – unadjusted; p_2_ – adjusted, additive model; p_3_ – adjusted, dominant model. AUC – area under the curve; HOMA-IR – homeostasis model assessment of insulin resistance; ISI – insulin sensitivity index; MAF – minor allele frequency; SNP – single nucleotide polymorphism. *subgroup (N = 491).

**Figure 1:**
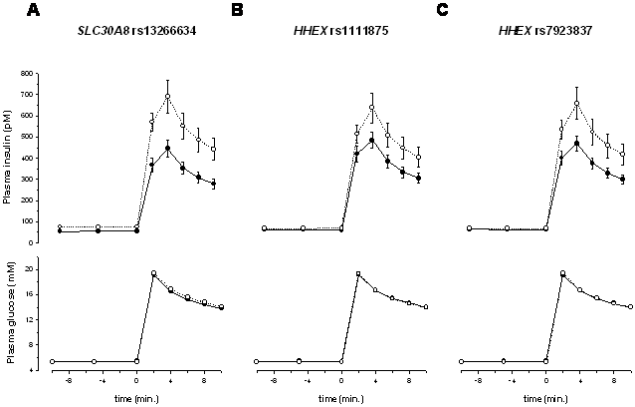
Association of *SLC30A8* SNP rs13266634 (A) and *HHEX* SNPs rs1111875 (B) and rs7923837 (C) with glucose-stimulated insulin secretion. Plasma levels of glucose (lower panels) and insulin (upper panels) during IVGTT. Dominant model: black circles – homozygous carriers of the major allele; white circles: heterozygous and homozygous carriers of the minor allele.

To assess the effects of the candidate SNPs specifically on glucose-stimulated insulin secretion, we analysed data obtained from an IVGTT in a subgroup of 150 subjects. The incremental AUC of insulin levels of *EXT2* SNP rs11037909 and *LOC387761* SNP rs7480010 were not significantly correlated with insulin secretion prior to (p = 0.9 and p = 0.8, respectively, dominant model) as well as after adjustment for gender, age, BMI, and clamp-derived insulin sensitivity (p = 0.8 and p = 0.6, respectively, dominant model). As presented in Figure 1, the major alleles of *SLC30A8* SNP rs13266634 and the *HHEX* SNP rs7923837 were significantly correlated with reduced insulin secretion prior to adjustment (p = 0.0029 and p = 0.0359, respectively, dominant model). After adjustment for gender, age, BMI, and clamp-derived insulin sensitivity, the *SLC30A8* SNP rs13266634 and the *HHEX* SNP rs7923837 remained significantly correlated with insulin secretion (p = 0.0416 and p = 0.0254, respectively, dominant model). *HHEX* SNP rs1111875 showed a trend towards an association with insulin secretion that however did not withstand adjustment (unadjusted p = 0.09, adjusted p = 0.16, dominant model). The pathophysiological importance of the *HHEX* SNP rs7923837 was further stressed by the significant correlation of its major allele with increased plasma glucose levels at 120 min. of OGTT (Table 3).

## Discussion

In summary, we did not detect associations of the recently reported T2DM candidate SNPs [14] with state-of-the-art measures of insulin sensitivity. The significant correlation observed between *SLC30A8* SNP rs13266634 and clamp-derived insulin sensitivity was not retained after adjustment for gender, age, and BMI, and thus might be due to this SNP's effect on (abdominal) adiposity. Furthermore, neither the representative *EXT2* SNP rs11037909 nor the *LOC387761* SNP rs7480010 were associated with insulin secretion, as assessed by OGTT and IVGTT. Moreover, neither *EXT2* nor *LOC387761* could be confirmed as T2DM genes in four very recently published genome-wide association studies [9], [18]–[20]. Thus, further studies that should include the analysis of the complete genetic variation within these loci are needed to finally clarify these genes' roles in the pathogenesis of T2DM.

In contrast, we observed clear associations of SNP rs7923837 within the *HHEX* locus with insulin secretion indices derived from OGTT as well as with glucose-stimulated insulin secretion during the IVGTT. Even though our statistical data were not corrected for multiple comparisons, this SNP's association with measures of insulin secretion derived from two independent methods (OGTT and IVGTT) largely excludes the possibility of a by-chance finding. Moreover, the association of SNP rs7923837 with plasma glucose levels at 120 min. of OGTT indeed suggests a very important role of the *HHEX* gene in the development of impaired glucose tolerance and T2DM. This is further strengthened by the aformentioned genome-wide association studies all of which confirmed the importance of *HHEX* as a T2DM gene [9], [18]–[20]. As already pointed out by Sladek et al. [14], *HHEX*'s role in the predispostion to T2DM could be attributed to this gene's function during organogenesis of the ventral pancreas [21], [22].

At the moment, we cannot explain the discrepancy between the association of *SLC30A8* SNP rs13266634 with glucose-stimulated insulin secretion during the IVGTT and the lack of association with OGTT-derived parameters for insulin secretion. However, this gene's function as a zinc transporter in secretory vesicles of pancreatic β-cells providing zinc for insulin maturation and/or storage [23], [24] renders this gene a very plausible candidate for β-cell dysfunction. In addition, *SLC30A8* was replicated as a T2DM gene in three out of four of the recently reported genome-wide association studies [18]–[20].

In conclusion, genetic variants of the *HHEX* and *SLC30A8* loci are associated with altered glucose-stimulated insulin secretion. Therefore, the major alleles of candidate SNPs within these loci represent crucial alleles for β-cell dysfunction and, thus, might confer increased susceptibility of β-cells towards adverse environmental factors.
